# Renal cell cancer without a renal primary

**DOI:** 10.1186/1477-7819-8-18

**Published:** 2010-03-22

**Authors:** M Wayne, W Wang, J Bratcher, B Cumani, F Kasmin, A Cooperman

**Affiliations:** 1 Pancreatic and Biliary Center N.Y, 170 W12th St., Cronin Bldg, NY, NY 10011, USA

## Abstract

Renal cell carcinoma has been increasing in incidence over the past two decades. Men are affected more than women and metastatic disease at presentation occurs in up to one third of patients. Metastasis can occur to virtually any organ, and involvement of multiple organs is not uncommon. To date, no reports have been found of metastatic disease without a renal primary. We present a case of renal cell cancer initially presenting as a subcutaneous mass with subsequent pancreatic and parotid gland metastases in absence of a primary renal source.

## Background

In the United States, renal cell carcinoma (RCC) has incidence in excess of 30,000 cases, with 12,000 deaths every year from the disease [1]. It occurs predominantly in males in their sixth to eight decade of life, and African Americans have a 10-20% higher incidence [2]. RCC is well known for its ability to metastasize to nearly every organ system of the body. Metastasis usually occurs several years after identification of the renal primary, but up to 30% of patients have metastatic disease on initial presentation [3]. The most common targets for metastases are lung, bone, lymph nodes, adrenal glands, brain, liver, and contralateral kidney [4]. In contrast, pancreatic and cutaneous involvement is exceedingly rare, occurring approximately 0.25-3% and 3.3% of the time, respectively [5]. Metastatic RCC is typically classified as either synchronous (detected at the same time as primary tumors) or metachronous (detected after a time interval from primary tumor, normally >6 months). In fact, it is not uncommon for metastatic pancreatic lesions to develop several years after nephrectomy [6]. RCC with pancreatic involvement can be a diagnostic challenge in differentiating between primary pancreatic cancer and metastatic disease. Our case exemplifies this diagnostic difficulty as the patient developed subcutaneous, pancreatic and parotid gland metastatic foci of RCC without ever having developed evidence of a renal primary.

## Case presentation

In October, 2007 a 61-year-old woman presented to Saint Vincent's Medical Center with a 5 cm subcutaneous growth on her left upper extremity. Histological examination after surgical excision of the mass revealed a clear cell neoplasm consisting of polygonal cells with abundant clear cytoplasm, containing faint granular material. Immunohistochemical analysis demonstrated positive CD10 and AE1/AE3 staining. Pathologic interpretation of the mass was highly suggestive of metastatic RCC of the clear cell type. There were no lesions present anywhere else by physical examination or CT scan.

The patient was closely followed in an attempt to locate a primary renal source of disease with multiple imaging studies negative for a renal primary or other sites of metastasis. However, repeat CT scan 9 months later revealed an asymptomatic pancreatic mass. Endoscopic evaluation was performed with endoscopic ultrasound and fine needle aspiration (EUS/FNA). The study demonstrated a 2-cm hyperechoic, well-defined lesion in the body of the pancreas. The remaining pancreatic parenchyma was otherwise normal without ductal dilation or evidence of pancreatitis. Histomorphological analysis of the core biopsy samples yielded similar findings to those of the upper extremity mass. Additionally, an immuno-profile was focally strong for both CD10 and PNRA, which was again highly suggestive of renal cell carcinoma. A central pancreatectomy was performed in August 2008 and tissue samples were positive for PRNA, Vimentin, and CD10, correlating strongly with RCC. The patient continued periodic surveillance to identify a renal primary and further metastasis at three month intervals. Six months later, physical exam revealed left parotid gland enlargement and an MRI revealed a 1.6 cm enhancing mass in the left parotid gland. No other lesions were found on surveillance PET/CT scan at that time. The patient had a superficial parotidectomy and again, pathological analysis demonstrated a clear cell carcinoma that was identical to the previous subcutaneous and pancreatic specimens. The sample was sent for expert verification at an outside institution, which corroborated our findings. Currently, the patient is doing well and is undergoing surveillance at 6 month intervals. To date, a renal primary has not been found.

## Conclusions

RCC has an annual incidence in excess of 30,000 cases in the United States and its greatest incidence occurs in males during the sixth decade of life [7]. The nature of this tumor distinguishes itself from other cancers in several respects. Namely, it's peculiar ability to metastasize to nearly every region of the body several years after initial presentation. It also differs from other neoplasms in its predilection for both hematogenous and lymphatic spread. We present the first recorded case of three metastatic foci without the identification of a renal primary, one of which mimicked a primary pancreatic neoplasm. As far as pancreatic cancers are concerned, metastatic tumors comprise about 3% of pancreatic tumors overall [7]. Initial clinical symptoms include abdominal pain, weight loss, fatigue, anemia, diarrhea, and jaundice. However, mass lesions often do not produce any recognizable symptoms and are only diagnosed when found incidentally on radiographic imaging, as was the case in our report. Furthermore, solitary pancreatic metastasis from RCC can mimic primary pancreatic neoplasms, including pancreatic neuroendocrine tumors (islet cell tumor), solid pseudopapillary tumors, mixed ductal-endocrine carcinomas, ductal adenocarcinomas with clear cell features, perivascular epithelioid cell tumors (sugar tumor), or solid serous cystadenomas [8,9]. Most notably RCC and clear cell primary tumors of the pancreas may show considerable overlap in both clinical setting and pathological appearance, making complete distinction between the two tumors very difficult without additional studies. Morphologically, pancreatic ductal adenocarcinoma with clear cell features are composed of pleomorphic cells with abundant clear cytoplasm and well-defined cell borders. Nuclei are moderately pleomorphic with irregular borders and often eccentrically positioned. Chromatin varies from vesicular to coarsely granular and nucleoli are not prominent. Alternatively, nuclei of RCC tend to be round and uniform, with finely granular, evenly distributed chromatin. Depending upon the degree of differentiation, nucleoli may be absent, sparse, large, or prominent. Occasionally, there are very large nuclei lacking nucleoli or bizarre nuclei [10]. The architectural growth patterns of clear cell RCC can vary, ranging from sinusoidal and sheet-like solid patterns to alveolar, tubular, or acinar appearances. No luminal differentiation is apparent in the alveolar pattern but a central, rounded luminal space filled with lightly acidophilic serous fluid or erythrocytes occurs in the acinar pattern. Infrequently, clear cell RCC has a distinct tubular or tubulopapillary architecture.

Immunohistochemical studies are helpful to distinguish metastatic from primary pancreatic tumors. According to the literature, 90-100% of pancreatic adenocarcinomas express CK7 as well as CK8,13,18, and 19 [11-13]. Even though CK20 is found in less than 20% of pancreatic cancers, most studies report the CD7+/CK20+ as the most common and the CK7+/CK20- as the second most common staining patterns [14], although the reverse has also been reported [11]. The coordinate staining pattern CK7-/CK20+ was found in up to ten percent of pancreatic ductal adenocarcinomas. Glycoprotein tumor antigens CEA and CA19-9 are reported to be positive in a variety of patients with pancreatic adenocarcinoma. As for clear cell RCC, lack of both CK7 and CK20 expression has been found [15-17], although papillary and chromophobe RCC were reported to have some CK7 expression [18-20]. CEA has been reported to be negative in all metastatic clear cell RCC to the pancreas [7]. Finally, vimentin staining is normally positive in RCC, occurring in >90% of cases, while it is non-reactive in more than 90% of pancreatic adenocarcinomas [21].

For patients with solitary pancreatic metastases, surgical treatment should be recommended because it is more effective than other treatments such as radiation and chemotherapy. The mean survival reported in the literature is only 1.3 years following metastatic focus resection [7], but 5 year survival rates as high as 68% have been documented [22].

In summary, this case describes the first documented report of metastatic RCC without the determination of a primary renal tumor. In the vast majority of cases, the primary renal lesion is found through subsequent radiographic surveillance, sometimes up to several years after the discovery of the initial metastatic lesion, which did not occur in our patient. The fact that this case presented with evidence of metastasis to three different sites (the subcutaneous tissue of the arm, the pancreas and the parotid gland) demonstrates the interesting ability of RCC to invade almost any organ and also reinforces the need for a thorough work-up to distinguish primary pancreatic neoplasm from another metastatic process. Although many cases are found incidentally from radiographic imaging obtained for other reasons, tissue sampling for pathological and immunohistochemical analysis is essential to help determine the tumor origin. When metastatic RCC is found, complete surgical resection should be the treatment of choice when medically feasible.

## Competing interests

The authors declare that they have no competing interests.

## Consent

Informed Consent was obtained from the patient for the publication of this case report.

## Authors' contributions

MW is the lead author and surgeon for this case. It is his patient being written about.

WW is the lead pathologist, who did extensive research on this case. BC is the resident doctor who performed the literature search. JB is the GI doctor who aided in the review and correction of this article. FK is the GI doctor who performed the procedure. AC is the senior surgeon who assisted in the case and in the write up of the case.
